# Automatic segmentation of inferior alveolar canal with ambiguity classification in panoramic images using deep learning

**DOI:** 10.1016/j.heliyon.2023.e13694

**Published:** 2023-02-11

**Authors:** Shuo Yang, An Li, Ping Li, Zhaoqiang Yun, Guoye Lin, Jun Cheng, Shulan Xu, Bingjiang Qiu

**Affiliations:** a Center of Oral Implantology, Stomatological Hospital, Southern Medical University, Guangzhou, China; b Guangdong Provincial Key Laboratory of Medical Image Processing, School of Biomedical Engineering, Southern Medical University, Guangzhou, Guangdong, China; c National-Regional Key Technology Engineering Laboratory for Medical Ultrasound, Guangdong Key Laboratory for Biomedical Measurements and Ultrasound Imaging, School of Biomedical Engineering, Health Science Center, Shenzhen University, Shenzhen, China; d Department of Radiology & Guangdong Cardiovascular Institute & Guangdong Provincial Key Laboratory of Artificial Intelligence in Medical Image Analysis and Application, Guangdong Provincial People's Hospital, Guangdong Academy of Medical Sciences, Guangzhou, China; e Data Science Center in Health (DASH) & 3D Lab, University Medical Center Groningen, University of Groningen, Groningen, Netherlands

**Keywords:** Deep learning, Convolution neural network (CNN), Image segmentation, Inferior alveolar canal (IAC), Panoramic radiography (PR)

## Abstract

**Background:**

Manual segmentation of the inferior alveolar canal (IAC) in panoramic images requires considerable time and labor even for dental experts having extensive experience. The objective of this study was to evaluate the performance of automatic segmentation of IAC with ambiguity classification in panoramic images using a deep learning method.

**Methods:**

Among 1366 panoramic images, 1000 were selected as the training dataset and the remaining 336 were assigned to the testing dataset. The radiologists divided the testing dataset into four groups according to the quality of the visible segments of IAC. The segmentation time, dice similarity coefficient (DSC), precision, and recall rate were calculated to evaluate the efficiency and segmentation performance of deep learning-based automatic segmentation.

**Results:**

Automatic segmentation achieved a DSC of 85.7% (95% confidence interval [CI] 75.4%–90.3%), precision of 84.1% (95% CI 78.4%–89.3%), and recall of 87.7% (95% CI 77.7%–93.4%). Compared with manual annotation (5.9s per image), automatic segmentation significantly increased the efficiency of IAC segmentation (33 ms per image). The DSC and precision values of group 4 (most visible) were significantly better than those of group 1 (least visible). The recall values of groups 3 and 4 were significantly better than those of group 1.

**Conclusions:**

The deep learning-based method achieved high performance for IAC segmentation in panoramic images under different visibilities and was positively correlated with IAC image clarity.

## 1 Introduction

Inferior alveolar canal (IAC) is an essential anatomical structure of the mandible and includes inferior alveolar arteries, veins, and sensory nerve fibers [1]. IAC is highly related to the diagnosis and treatment results of oral diseases such as mandibular nerve anesthesia, maxillofacial surgery, implant surgery, and root canal therapy of mandibular posterior teeth [[2], [3], [4]]. Panoramic radiography (PR) is widely used to diagnose and treat dental diseases to effectively avoid IAC injuries when implementing treatment plans [5]. However, IAC often appears ambiguous and boundaries are blurred in PR images because of limitations in panoramic imaging technology and thin or missing of the osseous wall surrounding IAC. Furthermore, there is a large variation among patients, IAC is often invisible in PR images, further making IAC recognition or segmentation a challenging task. Moreover, IAC becomes invisible in a panoramic radiograph when the blood vessels and nerve plexus wrap around the IAC wall [6]. It has been reported that IAC is clearly delineated in PR in only 23.6% of the cases [7]. Nahar et al. divided the visibility of IAC in panoramic images into four levels with no good visibility in approximately 60% of cases [8,9]. The canal was entirely invisible in 32% (19/60) of patients (Naitoh et al., 2009). Poor visibility of IAC in panoramic images can be caused by various factors, including PR equipment and the position of patients’ IAC. Some studies have even observed a negative trend between the age of the patient and the visibility of IAC, which can be attributed to the reduction in bone density with age [10]. Therefore, manual IAC segmentation requires a large amount of expertise.

The increasing demand for oral health has led to large workloads for dentists using PR imaging, necessitating considerable time and labor. With advances in artificial intelligence, deep-learning methods have demonstrated tremendous performance in image segmentation [11,12]. Currently, machine assistants can potentially help dentists in clinical workflows. Deep learning techniques provide more flexible and powerful capabilities than traditional machine learning methods, and require less expert analysis, facilitating extension to other segmentation tasks. This can help expedite workflows and ultimately reduce overall costs. Recent studies have focused on tooth segmentation in panoramic radiography [13]. Several studies have reported deep-learning models that achieve radiologist-level performance on PR. Pandyan et al. reported that the deep learning method achieved high accuracy (92%) in identifying IAC, improving the Dice coefficient to 0.829 [14]. However, there are only a few traditional deep-learning models that perform automatic IAC segmentation [15]. Additionally, these models are not adopted on a large-scale because of two main factors: limited research results and lack of rigorous comparative evaluation studies on radiologists and automatic methods.

Previous studies have reported the use of deep learning methods to automatically segment IAC from PR images. However, the image quality has an evident impact on the IAC segmentation results. Evaluation of automatic IAC segmentation method in different image clarity is worth exploring. Furthermore, to our knowledge, no studies have studied in detail the deep-learning model and radiological visibility of IAC. The objective of this study was to: 1) adopt a popular deep neural network (EfficientUnet) to segment IAC in PR images and 2) investigate the performance of automatic segmentation under different IAC visibilities. Reference is made to Table 1 for abbreviations used throughout in this work.

**Table 1:** Definition of abbreviations.

Abbreviation	Definition
CI	Confidence Interval
CNN	Convolution Neural Network
DSC	Dice Similarity Coefficient
IAC	Inferior Alveolar Canal
PR	Panoramic Radiography

## 2 Materials and methods

### 2.1 Data collection and preprocessing

The dataset used in this study was obtained from the Stomatological Hospital of Southern Medical University, China. This study was approved by the Ethics Committee of the Stomatological Hospital of Southern Medical University, and was conducted according to the Declaration of Helsinki. The written informed consent was obtained from all patients. All patient-relevant information on PR images was anonymous and de-identified. Each PR image was captured by the same panoramic machine (V02.58.01, Sirona, Germany) under the same radiation conditions (64 KV, 16 mA, 14.1s). The patient's body position was strictly fixed during PR. Two senior radiologists with more than ten years of experience were invited to manually delineate IAC in all PR images using Mimics 17.0 software. Both radiologists' annotations of IAC were assembled as the ‘ground truth’ (GT) for both training and testing purposes. In this study, PR images of 683 patients were collected. All PR images and the corresponding IAC masks were resized to 512 × 256 pixels. Then, each PR image was evenly divided into two single images (left and right), where each image contained a complete IAC. Subsequently, the single image was cropped into two unilateral images of size 256 × 256 pixels. Finally, this division brought the total number of samples to 1366. The images were further normalized by transforming their gray values from the 0–255 range to 0–1. The data were randomly split into 1000 samples for training and 366 samples for testing.

### 2.2 Construction of the deep learning system

**Fig. 1:**
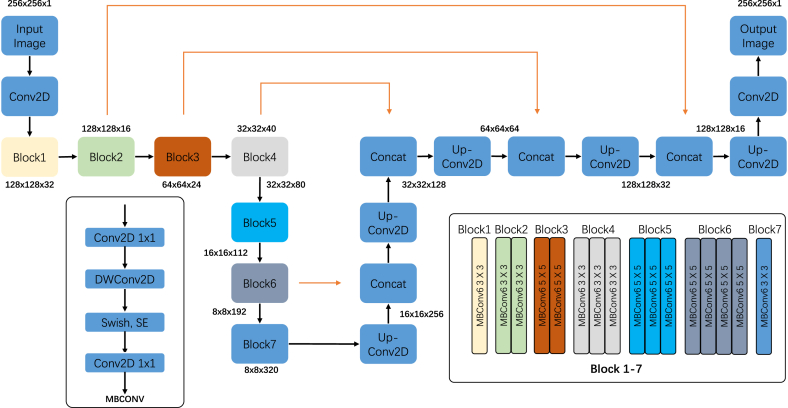
EfficientUnet architecture.

In this study, EfficientUnet [16,17] (Fig. 1) was adopted for the segmentation of IAC in PR images. Inspired by the success of Unet in medical image segmentation, EfficientUnet was designed as a U-net structured model consisting of an encoder and decoder. The encoder extracted representative downsampled features from the input images by performing repeated convolution and pooling operations. The decoder reconstructed high-resolution segmentation masks from the previous features by performing up-convolution operations. Each feature in the decoder was concatenated to the corresponding feature map in the encoder such that the number of feature channels was doubled. Thus, the network could propagate more contextual information to a higher-resolution layer.

During the training, horizontal and vertical translation, scale range, random rotation, tangent transformation, and horizontal inversion were performed as data augmentation to improve the generalization ability of the model. After reaching convergence, the well-trained Convolutional neural network (CNN) model was ready to be applied to the automatic segmentation of IACs in different types of oral medical images.

### 2.3 Dataset grouping

**Fig. 2:**
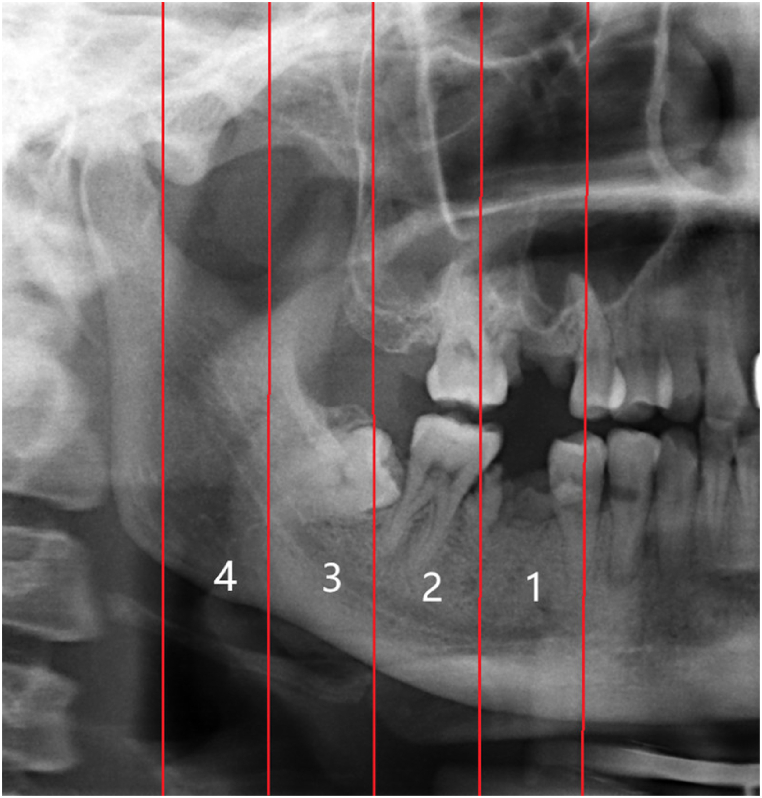
Quartering IAC from the mental foramen to the mandible foramen.

**Fig. 3:**
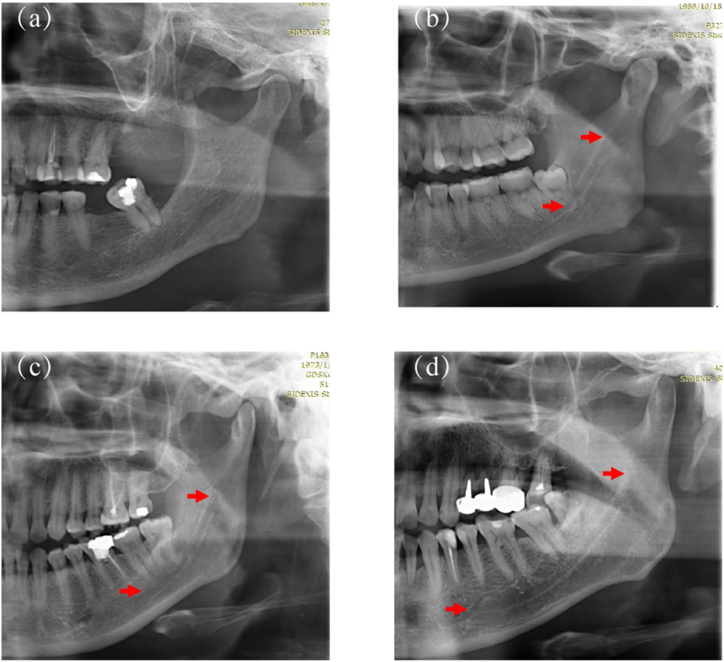
Validation dataset group for different degrees of IAC visibility: (a) the entire IAC is invisible; (b) ≦1/2 IAC is clearly visible; (c) ≧1/2 IAC is clearly visible; and (d) the entire IAC displays good visibility.

Inspired by the studies from Kamrun [8] and Angelopoulos [18], the number of groups was set to 4. The testing dataset (n = 366) was divided into four groups based on image visibility to assess the performance of the CNN model and further compare it with the performance of two experienced radiologists. In a left/right PR image, IAC was bisected into four segments from the mental foramen to the mandibular foramen on average, defined as regions 1–4, respectively, as shown in Fig. 2. Based on the visibility of the four IAC regions, the validation dataset was grouped into: Group 1—the entire IAC is invisible (Fig. 3a); Group 2—less than half of IAC is clearly visible (Fig. 3b); Group 3—more than half of IAC is clearly visible (Fig. 3c); and Group 4—the entire IAC is clearly visible (Fig. 3d). One radiologist with more than ten years of experience manually grouped the PR images. The grouping was further reviewed and revised by a second radiologist with more than ten years of clinical experience in dentistry. A third radiologist was invited to settle the difference when the groupings of the two radiologists were not consistent.

### 2.4 Experimental conditions

In this study, a deep learning framework was implemented using Keras with a TensorFlow backend. The experiments were performed on a workstation equipped with Intel i7-8086 k CPU and GTX1080Ti GPU. The batch size was set to 6 and Adam optimization with a learning rate of 0.001 was used to update the parameters during training. The total number of training epochs was set to 200 and the network training converged in more than 3 h. Furthermore, an early stopping strategy was utilized if there was no improvement in the loss of the validation set after ten epochs to avoid overfitting.

### 2.5 Calculation and analysis

After a thorough training, Groups 1–4 of the testing dataset were fed into the EfficientUnet model to obtain automatic segmentation masks for IAC. Manual annotations from the two radiologists each with more than ten years of experience were used as the GT. The average segmentation time per image of the automatic model and radiologists was used to evaluate the efficiency of the automatic segmentation model. The Dice similarity coefficient (DSC), precision, and recall were used to assess segmentation performance. The DSC value is widely applied to measure the consistency between automated segmentation and GT. Precision describes the purity of optimistic predictions relative to GT and recall represents the completeness of the optimistic predictions with respect to GT. DSC, precision, and recall are defined as follow: $DSC=\frac{2TP}{2TP+FP+FN}$, $precision=\frac{TP}{TP+FP}$, and $recall=\frac{TP}{TP+FN}$, which can be obtained by four indicators: true positive (TP), false positive(FP), false negative (FN) and true negative (TN). The student's t-test was used to compare the means of DSC, precision, and recall values for the four groups. The significance level was set to p < 0.05. SPSS 20.0 was used for all statistical analyses in this study.

## 3 Results

The test dataset (*n* = 366) was divided into four groups. The images in Group 1 had no IAC visibility, accounting for 18.9% (69 images) of the entire testing dataset. Groups 2 and 3 were most likely the cause of disagreement between the two radiologists during grouping, containing 140 and 100 images, respectively, and accounted for 65.5% of the testing dataset. A third radiologist intervened for the 10 images with different grouping opinions divided into Groups 2 and 3. The images in Group 4 had good visibility of IAC, accounting for 15.6% (57 images) of the testing dataset. This study found that for the testing dataset, the average time spent by radiologists to manually annotate IAC was 5.9 s (range to 3.7–9.5 s) per image, whereas the average time of the automatic IAC segmentation model was just 33 ms per image. This means that the CNN model was significantly faster than manual annotation (*t*-test: p < 0.05).

The segmentation performance of our CNN model was further evaluated using DSC, precision, and recall as evaluation metrics. The average DSC, precision, and recall of our model were higher than 80%, with values of 85.7% (75.4–90.3), 84.1% (78.4–89.3), and 87.7% (77.7–93.4), respectively. For Group 1, the average DSC, precision, and recall values were 82.7% (75.4–88.3), 80.0% (78.4–83.3), and 86.2% (77.7–89.4), respectively. For Group 2, the average DSC, precision, and recall values were 85.3% (81.4–87.6), 84.1% (81.2–86.3), and 87.3% (83.7–89.4), respectively. For Group 3, the average DSC, precision, and recall values were 87.2% (82.4–88.3), 86.0% (81.3–87.1), and 88.8% (84.0–91.4), respectively. For Group 4, the average DSC, precision, and recall values were 89.1% (85.4–90.3), 88.0% (85.4–89.3) and 90.1% (87.7–93.4) (Table 2). These results demonstrated that the CNN model was robust and accurate enough to automatically annotate IAC in PR images.

**Table 2:** Validation dataset group average DSC, precision and recall value for IAC segmentation performance of CNN model.

	DSC, % (95% CI)	Precision, % (95% CI)	Recall, % (95% CI)
Group1 (n = 69)	82.7* (75.4–88.3)	80.0* (78.4–83.3)	86.2** (77.7–89.4)
Group2 (n = 140)	85.3 (81.4–87.6)	84.1 (81.2–86.3)	87.3 (83.7–89.4)
Group3 (n = 100)	87.2 (82.4–88.3)	86.0 (81.3–87.1)	88.8** (84.0–91.4)
Group4 (n = 57)	89.1* (85.4–90.3)	88.0* (85.4–89.3)	90.1** (87.7–93.4)

Note: * means that the average DSC and precision values of Group 4 were significantly higher than those of Group 1 (p < 0.05).

** means that the average recall values of Groups 3 and 4 were considerably more extensive than those of Group 1 (p < 0.05).

In the above results, it was observed that Group 4 displayed the best performance, while Group 1 had the lowest matching degree in the radiologists’ opinions. For pairwise comparisons between different groups, the average DSC and precision values of Group 4 were significantly higher than those of Group 1 (p < 0.05). The average recall values of Groups 3 and 4 were considerably more extensive than those of Group 1 (p < 0.05). These comparison results indicated that as the visibility of IAC improved, the DSC, precision, and recall values also improved. They also revealed that the higher the IAC visibilities in PR images, the closer the automatic segmentation results were to the manual annotations of the experienced radiologists.

## 4 Discussion

With the development of deep learning in precision medicine, it is becoming increasingly important to develop a reasonable diagnosis and treatment plan using various digital dental data [15]. As one of the current state-of-the-art machine learning methods, deep learning can achieve higher work efficiency and accuracy in related fields, and has become a hot research topic in clinical applications. The detection of IAC in PR images is important for the diagnosis and treatment of dental diseases and oral surgery [[19], [20], [21], [22]]. Modern algorithms that can be clinically applied for automatic IAC segmentation should be both fast and accurate. CNN models have the potential to satisfy these requirements.

The traditional detection of IAC relies mainly on experienced radiologists, while the detection efficiency and accuracy are highly related to the working time and experience of radiologists [23]. However, with the increase in digital oral image data, manual segmentation of IAC can no longer meets the increasing clinical demand. Long hours of intense work can lead to larger human bias. In this study, the average time required for the manual annotation of IAC was approximately 6s per image. For several images in which IAC displayed low visibility, the manual annotation time was longer than 10 s per image. In contrast, the average time of automatic segmentation using our well-trained EfficientUnet model was much shorter at just 33 ms per image. All comparison results in this study demonstrated that the efficiency of deep learning for IAC segmentation was significantly higher than that of manual annotation. With the improvement in computer hardware performance and deep learning techniques, the operation speed of deep learning-based auto-segmentation approaches will be higher while avoiding human bias and fatigue. This is important for clinical applications.

Currently, CNN models are widely used in medical image segmentation and diagnosis. Deep learning models can accurately localize and segment colorectal cancer in the magnetic resonance imaging images of most patients [24,25]. A coupled shape model in combination with a neural network was used to segment teeth in PR images and obtained good segmentation results [26]. In addition, a three-dimensional CNN model was proposed to automatically segment head and neck in CT images, achieving an average DSC coefficient of 0.793. Ibragimov and Xing applied a CNN model for the automatic segmentation of the mandible and obtained an average DSC of 89.5% [27]. All these studies demonstrate the excellent performance of CNNs in the segmentation tasks of oral medical images.

The comparison between automated segmentation and manual annotation demonstrated that the IAC position was highly consistent between the two segmentation approaches, with a matching degree close to 85% (Fig. 5). These results demonstrated that CNN models have the potential to meet daily clinical needs. The edges of IAC predicted by the CNN model were more accurate than those predicted by manual delineation. In some special cases such as posterior maxillary area implants, posterior tooth root canal therapy, and even the postoperative review of the mandible, the location of IAC could be accurately distinguished by the CNN model.

The visibility of IAC in PR images varies significantly among different individuals. Monsour reported that 36% of IACs in the area of premolar and molar could not be clearly observed [28]. Naitoh reported that only 32% of IACs in the molar area were clearly visible [9]. In Lindh's study, nearly 60% of IACs in the upper margins displayed poor visibility [7]. The visibility of IAC in the posterior region was significantly better than that in the anterior region (Naitoh et al., 2009). Only 17% of the IACs in the validation dataset were clearly visible. In addition, the visibility of IAC among the elderly and women was worse than that of other segments of the population, which might be related to the decrease in bone mineral density. These results are consistent with findings in the literature.

**Fig. 4:**
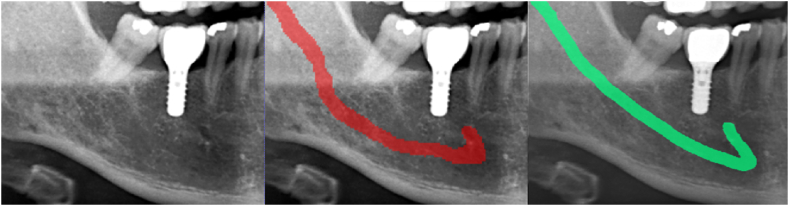
Representative automatic and manual annotation examples for Group 1. From left to right: Original PR image, Automatic annotation (CNN) and Manual annotation (ground truth). The red color indicates automatic segmentation of IAC, while the green color indicates manual annotation. (For interpretation of the references to color in this figure legend, the reader is referred to the Web version of this article.)

**Fig. 5:**
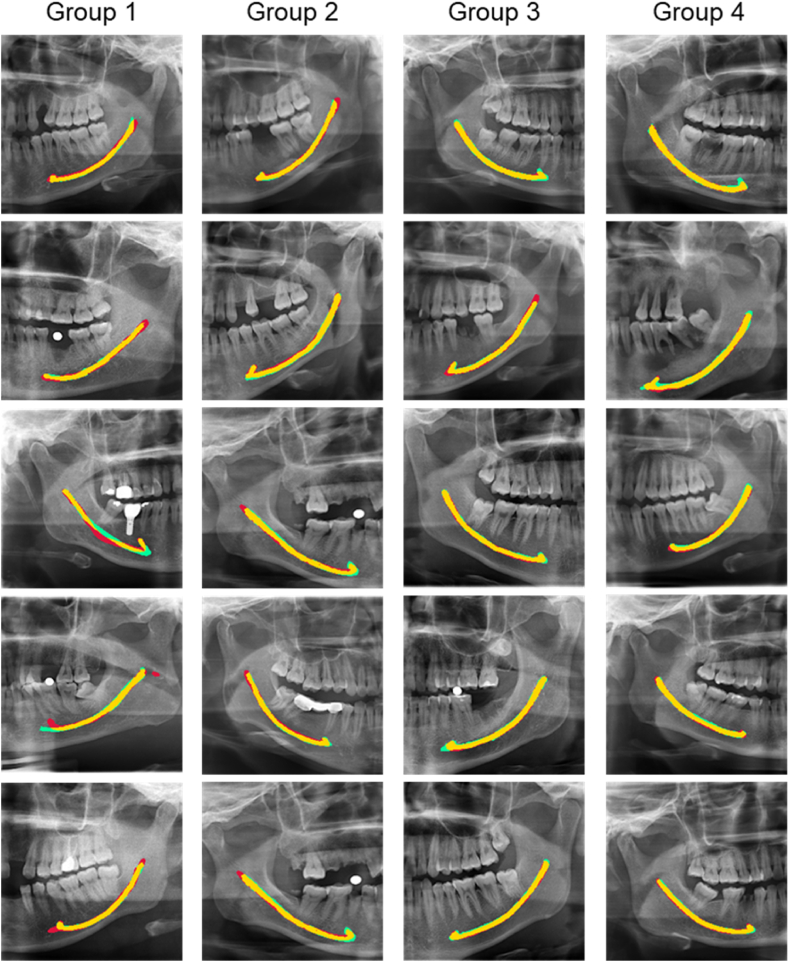
Comparison of partial segmentation results for automated segmentation and manual annotation. From left to right: Group 1, Group 2, Group 3 and Group 4. The red color indicates automatic segmentation of IAC, while the green color indicates manual annotation. Yellow represents the overlap of automated and manual annotations. (For interpretation of the references to color in this figure legend, the reader is referred to the Web version of this article.)

In this study, to better compare the performance of our CNN model and manual annotation, a new quantitative grouping method was adopted to evaluate the segmentation results of IAC in panoramic images under different visibility conditions. To the best of our knowledge, this is the first study to use such a specific grouping approach for the evaluation of IAC segmentation. All images were divided into four groups based on the visibility of IAC and the evaluation metrics were calculated for each group. The overall degree of match between the CNN model and manual method was satisfactory, but there were still differences in the segmentation results of each group. For images in Group 1 where the visibility of IAC was low, there were five images with different annotations from the two radiologists, requiring an additional radiologist to make the final call. Most segmentation predictions from the CNN model were similar to those of the radiologists. However, for some images with inaccurate predictions, especially for those in which the IAC boundary could not be discerned, there was a big difference between automated segmentation and manual annotation (Fig. 4). Some upper edges of the IACs were discontinuous in Groups 2 and 3. In such cases, radiologists can only delineate IACs by subjective experience, whereas the CNN model performed better because of its ability in extracting weak feature structures. In the area of the premolars, manual annotation slightly outperformed the CNN model. The match between manual annotation and CNN prediction was the highest for Group 4, with each metric being more significant than 88%. All these results show a positive correlation between the clear delineation of PR images and CNN performance. In Groups 1 to 4, with increased area and clarity range of IAC, the segmentation results of the two methods matched more closely (Fig. 5). Data quality can substantially influence model performance.

This study first analyzed the impact of different image visibilities of IAC on automatic segmentation results. Obtaining a robust and accurate CNN segmentation model requires manual annotation of data, which constitutes a significant amount of work. The accuracy of the segmentation results was also affected by the quantity and quality of the data samples and manual delineation. At present, some research uses a generative adversarial network as a semi-supervised approach in medical image segmentation [8]. Our future work will focus on employing semi-supervised learning methods to reduce the cost of manual labelling. Furthermore, the panoramic radiography technique plays a vital role in a screening examination. The clinicians don't confirm the IAC status not only in PR but also in CT or CBCT. If the current AI model improved, severe IAC status should be verified using CT or CBCT.

In this study, panoramic images were the source of data. Although PR imaging is most commonly employed in dental clinical work, it may contain deformation and reconstruction errors due to the imaging principle of two-dimensional images. These deformations and errors result in the low visibility of IAC in the PR image, which poses a great challenge to both manual and automatic segmentation. In future studies, this limitation will be addressed in two ways. First, the CBCT image was used as the data source for the segmentation of IAC. The CBCT images, which are three-dimensional, can provide more comprehensive information on IAC without the problem of low visibility, benefitting segmentation performance. Second, the CNN model can be further optimized to make it more robust for IAC segmentation when visibility is low. Because the manual annotations of blurred images may not be sufficiently accurate, a new low-visibility training dataset will be constructed by adding random noise to high quality images while keeping the IAC masks unchanged. This reconstructed training dataset will help to improve the segmentation accuracy on blurred images. Furthermore, adding prior knowledge of the IAC shape to the loss function should improve the segmentation results. In reality, the performance of a deep learning model in real tasks is closely related to the given source data. Our dataset may be limited and cannot sufficiently represent the general population in clinical practice. In that regard, future research will focus on validation of the auto-segmentation model to other PR datasets.

## 5 Conclusion

The performance of the EfficientUnet model for IAC segmentation improved as the visibility of the panoramic image increased. All the segmentation results showed that EfficientUnet can be used in the daily diagnosis and treatment of oral diseases and can help reduce radiologists’ workload.

## Credit author statement

Bingjiang Qiu: Conceived and designed the experiments; Analyzed and interpreted the data; Wrote the paper.Shuo Yang: Conceived and designed the experiments; Performed the experiments; Analyzed and interpreted the data; Contributed reagents, materials, analysis tools or data; Wrote the paper.An Li: Performed the experiments; Analyzed and interpreted the data; Contributed reagents, materials, analysis tools or data; Wrote the paper.Ping Li: Performed the experiments; Analyzed and interpreted the data; Contributed reagents, materials, analysis tools or data.Zhaoqiang Yun: Analyzed and interpreted the data; Contributed reagents, materials, analysis tools or data.Guoye Lin: Conceived and designed the experiments.Jun Cheng: Contributed reagents, materials, analysis tools or data.Shulan Xu: Contributed reagents, materials, analysis tools or data; Wrote the paper.

## Fundings

The present study was supported by the 10.13039/501100021171GuangDong Basic and Applied Basic Research Foundation, China (No. 2021B1515120059) and 10.13039/501100009330Medical Scientific Research Foundation of Guangdong Province, China (No. A2020458).

## Declaration of competing interest

The authors declare that they have no known competing financial interests or personal relationships that could have appeared to influence the work reported in this paper.
